# Interactome of Paraoxonase PON2 Reveals New Pathways for Tumor Growth Regulation

**DOI:** 10.1134/S1607672922700089

**Published:** 2023-01-18

**Authors:** V. D. Karlov, N. B. Pestov, M. I. Shakhparonov, T. V. Korneenko

**Affiliations:** 1 Shemyakin-Ovchinnikov Institute of Bioorganic Chemistry, Moscow, Russia; 2 All-Russia Research Institute of Agricultural Biotechnology, Moscow, Russia; 3 Moscow Institute of Physics and Technology, Dolgoprudny, Russia; 4 Chumakov Federal Scientific Center for Research and Development of Immune-and-Biological Products of Russian Academy of Sciences, Moscow, Russia; 5 Institute of Biomedical Chemistry, Moscow, Russia

**Keywords:** interactome, paraoxonase, two-hybrid yeast screening, PON2, PPP4R2, ACAA2, DCN, CFAP53/CCDC11, SFRP4, ITM2A

## Abstract

The interactome of paraoxonase-2 encoded by the PON2 gene was investigated. A cDNA library was screened using a yeast two-hybrid system to search for new proteins interacting with human PON2. Analysis of the identified candidates, along with previously published data on interactors obtained by other methods, indicates the presence of a significant number of indirect interactions between PON2 and EGFR and, consequently, possible regulation of tumor growth with mutant EGFR involving PON2.

## INTRODUCTION

Historically, term “paraoxonase” was associated with the ability of mammalian blood plasma to enzymatically degrade paraoxon, chlorpyrophos-oxon, and many other organophosphates [1], and to some extent also catalyze the hydrolysis of the P-F bond in sarin and soman [2], providing protection against low doses of organophosphates, with the level of this protection varying significantly between individuals [3, 4]. Hydrolysis of diisopropyl fluorophosphate by human and rabbit blood plasma components was first discovered in 1946 [5], this report can be considered the first mention of these enzymes, and its dependence on Ca^2+^ was established. Currently, the family of mammalian paraoxonases includes paraoxonase 1, 2, and 3, (PON1, PON2, PON3), in humans the genes of all three enzymes are located in the long arm of the 7th chromosome (7q21.3-q22.1) [6, 7]. The PON1 and PON3 genes are present only in mammals, but a large number of aquatic species, especially those capable of deep diving, have lost the PON1 gene, reflecting their high sensitivity to some insecticides [8]. Paraoxonases are proteins with a mass of about 45 kDa, their three-dimensional structure is stabilized by one disulfide bond (one cysteine residue remains free), forms a six-bladed propeller with key histidine residues and bound calcium ions in the active center [9]. PON1 and PON3 are mainly synthesized in the liver and secreted into the blood [10]. PON2 differs significantly from its paralogs, it is expressed almost ubiquitously in all tissues, it is an intracellular membrane resident with a glycosylated [11] C-terminal ectodomain exposed outside the cell or inside the ER lumen, lysosomes, mitochondrial intermembrane space, or perinuclear space [12]. PON2 is not secreted into the blood plasma, but is secreted into the intestinal lumen, where it is important for resistance to infections [13, 14]. Paraoxonases are multifunctional enzymes [11, 15]: PON1 and PON3 are responsible for blood plasma paraoxonase and low specificity aryl esterase and lactonase activities, while PON2 in terms of substrate specificity should not be termed a paraoxonase [16]. In plasma, PON1 functions as part of high density lipoproteins, HDL, and the main function of the enzyme is considered to be lipolactonase activity, which helps to neutralize moderately oxidized lipoprotein [17], apparently by detaching free hydroxylated fatty acids through cyclization and opening of the lactones. In general, plasma paraoxonases have a rather broad specificity, hydrolyzing various lactones and acyclic esters (such as naphthyl acetate) [18].

Paraoxonases have notable potential in the fight against cardiovascular disease, PON1 gene knockout leads to the development of severe atherosclerosis [19]. Since PON1 is present in HDL, it protects low density lipoproteins, LDL, against oxidative stress, reducing formation of foam cells from macrophages. The same is true for PON2—knockout of the PON2 gene has similar physiological consequences [20], and for some time the main function of PON2 was considered to be antiatherogenic [28]. But in reality, PON2 poorly hydrolyzes most PON1 and PON3 substrates, but, in comparison to them, PON2 is thought to be able to hydrolyze quite well such substances as N-acylhomoserine lactone (N-AHL) [16], which, like pyocyanin, is a virulence factor of *Pseudomonas aeruginosa* (hereinafter, PAE). PON2 is most effective against N-AHL with a long chain (e.g., N-(3-oxodecanoyl)-homoserine lactone, 3OC12-HSL). Therefore, PON2 activity is thought to play a major role in inhibiting the quorum sensing of opportunistic bacteria, particularly PAE, which is extremely common in hospital-acquired infections. Knockout of the PON2 gene causes a decrease in resistance to PAE [21]. Moreover, PON2 and PON3 are important parts of innate immunity to PAE [22], exhibiting antioxidant and anti-inflammatory functions. Overexpression of PON2 and PON3 can prevent PAE pyocyanin activated free radical formation, activation of the NF-κB-signaling pathway, and increased IL-8 secretion, thereby reducing oxidative stress and inflammation [22]. PAE, in turn, tends to suppress the enzymatic activity of PON2 by releasing unidentified inhibitors [23]. Paradoxically, upon PAE infection and exposure to 3OC12-HSL, PON2 may serve a pro-apoptotic function that is associated with unidentified protein-protein interactions [24]. It is likely that in the absence of the formation of fully functioning complexes, PON2 becomes an easy target for inhibitors of bacterial nature, this inevitably leads to a more aggressive behavior of PAE and increased sensitivity of host cells to pyocyanin-type toxins, whose secretion is activated by quorum sensing.

Interestingly, PON2 expression is elevated in diseases of viral etiology, such as HIV infection [25, 26], but it is unknown whether this reflects activation of some resistance mechanism or, conversely, contributes to pathology. PON2 reduces caspase activation-induced endoplasmic reticulum stress [12].

The multi-functionality of PON2 is also illustrated by the disruption of insulin signaling when this gene is knocked out [27]. An extremely important feature of PON2—in contrast to the secreted PON1 and PON3 working in HDL—is that it performs “antioxidant” functions in the plasma membrane [28]. The protective function of PON2 under oxidative stress is also evident in the brain, with some polymorphisms showing an association with neurosensory hearing loss [29]. Here we would like to note that hearing impairment in children is also found in patients with a disrupted ATP8B1 gene (Byler disease) that, according to our data, is a protein partner of PON2 [30]. Unfortunately, the protective function of PON can also have negative consequences in certain cases, for example, in some cancer cell lines overexpression of PON2 increases cell resistance to chemotherapeutic agents and decreases tumor cell apoptosis [31]. Also, PON2 levels are elevated in vivo and in vitro in head and neck cancers, and the cells acquire greater resistance to radiotherapy [32]. In general, high PON2 levels in many cancers is a negative factor that decreases patient survival [33]. Importantly, there is evidence that the “antioxidant” function of PON2 may be unrelated to its lactonase activity [34]. Numerous conflicting data in favor of one or the other activity led us to continuation in PON2 interactome studies. The aim of our study was to find PON2 partner proteins, the identification of which will expand our understanding of the functional significance of this protein and possibly reveal new intracellular signaling pathways underlying pathophysiological processes in cancer.

## MATERIALS AND METHODS

**Yeast two-hybrid system.** Approaches similar to those previously published [35] were used. The search for PON2 interactors was performed with the Matchmaker® kit (Clontech, USA) and a cDNA library of human lung tissue. Two PON2 variants were cloned into the pGBKT7 plasmid (Clontech, USA): the CRA_a isoform (GenBank: EAW76763.1) with the leader sequence and another isoform without it (NCBI Reference Sequence: NM_001018161.2). The plasmids were transformed into yeast strain Y2HGold (“Clontech,” USA) by the lithium-acetate method. Then resulting clones were hybridized with Y187 strain carrying the cDNA library [35]. The first stage of selection was performed by growing on selective medium deficient in histidine, leucine, and tryptophan, i.e., three amino acids, which is important both to prevent plasmid loss and to induce a selective marker in the presence of interaction of the protein encoded by the cDNA of the screened library, i.e., with PON2. The grown colonies were then transferred to highly selective media providing a more stringent selection. Such regrowth was repeated several times to reveal any heterogeneity of the library and promote plasmid segregation. Plasmids were isolated from positive yeast clones and transformed into *E. coli* cells, and then purified and sequenced. The resulting sequences were used to identify the clones by analyzing sequence databases with BLAST. Several false-positive clones were then screened out by control cotransfection with plasmids carrying a control insert instead of PON2.

**Bioinformatic analysis of interactomes.** BioGRID (https://thebiogrid.org/), IntAct (https://www.ebi.ac. uk/intact/home), STRING (https://string-db.org/), GeneMania (https://genemania.org/), InBio Discover (https://inbio-discover.com/), HitPredict (http:// www.hitpredict.org/) databases were used both through their websites and through querying by Cytoscape 3.9.1. Interactors clusters were searched using the MCODE module.

## RESULTS AND DISCUSSION

We previously published the identification of new PON2 interacting proteins in A549 lung cancer cells using PON2 carrying a Halo-tag affinity tag at its C-terminus, followed by the identification of the PON2-bound proteins using LC-MS/MS [33]. However, the resulting list of associated proteins was apparently enriched with a large number of proteins that do not interact directly with PON2. Therefore, here we screened the cDNA library using a yeast two-hybrid system against two PON2 variants (with and without the leader sequence). The screening was successful only in the case of the full-length PON2. We identified several new partners: one clone showed interaction with decorin (DCN) isoform CRA_g (GenBank: EAW97458.1), two clones coding for integral membrane protein 2A (ITM2A, AAH40437.1), one clone with secreted frizzled-related protein 4 (SFRP4, NM_003014.4), acetyl-CoA acyltransferase (ACAA2, NM_006111.3) was detected in three clones, two clones with CFAP53/CCDC11 (NP_659457.2), and two clones of isoform 4 of the regulatory subunit 2 of protein phosphatase (PPP4R2, NP_001304956.1).

Unlike other PONs, the open reading frame of human and other primate PON2 have an N-terminal extension with no apparent homology to any other known sequences. It is possible that this peptide contributes to the formation of the PON2-specific interactome (since other PONs lack this cytoplasmic portion altogether).

In combination with the previously identified interaction of PON2 with ATP8B1, as well as all other interactors known from the literature and deposited into human protein interactome databases, a new version of the human PON2 interactome was constructed. Most of the data on PON2 interactions were obtained by high-throughput methods such as affinity capture or proximal biotin ligation followed by mass spectrometry. Both numerous high-throughput data (e.g., [36]) and our earlier data for PON2 [33] are characterized by a vast number of proteins identified as components of large and complex communities or even simply located in the same cellular compartment, while those that directly interact with the protein of interest remain unknown. In contrast, the yeast two-hybrid system is focused on identification of specifically binary interactions, but because of its unnatural yeast context it is also prone to interaction signals that may be either false positives or—to an even greater extent—false negatives. Therefore, any interactions need careful double-checking.

**Fig. 1.**
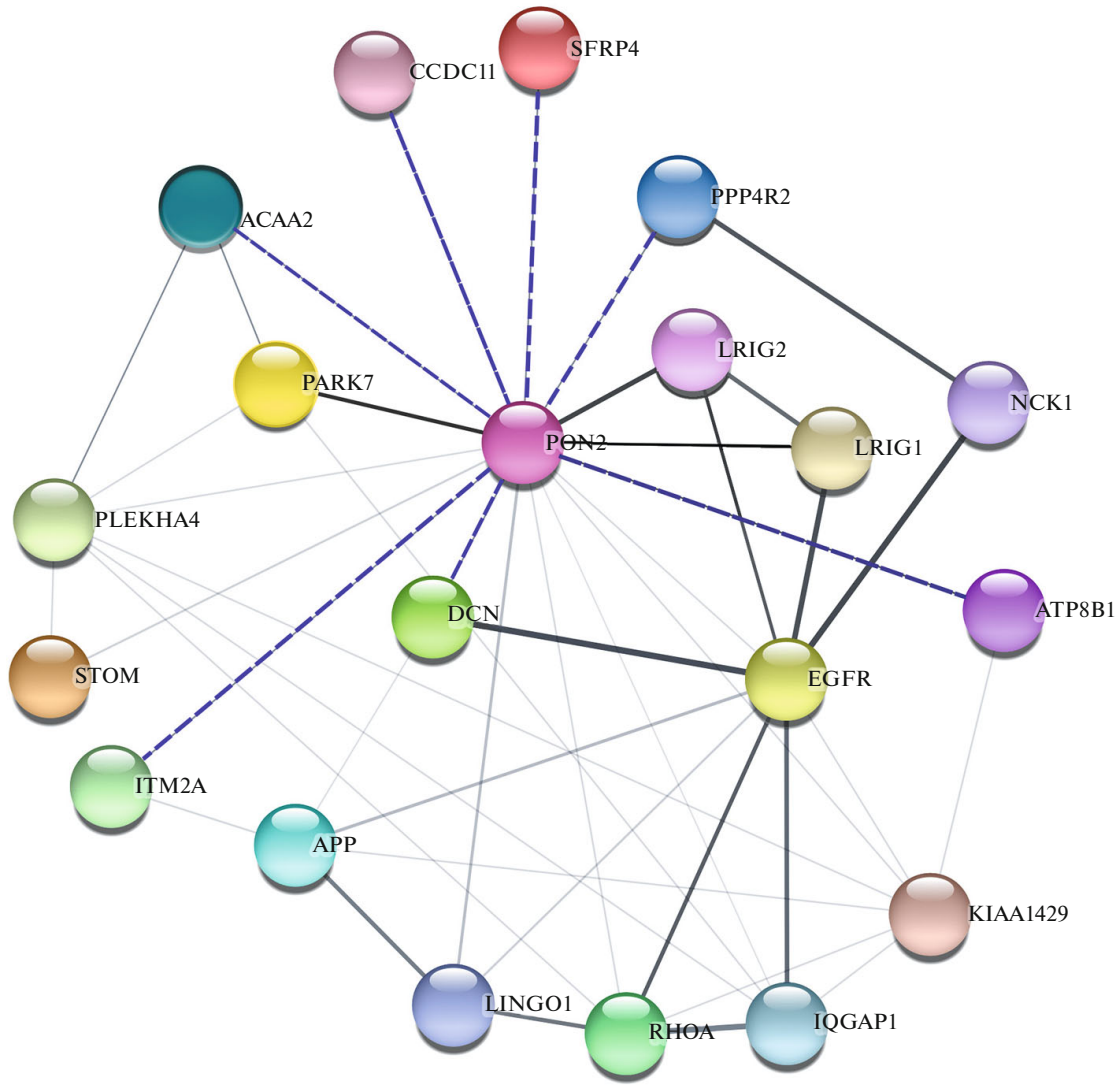
PON2 Interactome. High-confidence interactions are marked by bold lines. The interactions that we identified with the help of the two-hybrid system are shown by the purple dotted lines.

Unfortunately, only PARK7 (another name is DJ-1) [37] as well as LRIG1,2 [38] should currently be considered high-confidence PON2 interactions among human proteins. PON2 interaction with EGFR may also be regarded as a confirmed one, since it was found in several high-throughput studies (for example, [36]), but this may reflect the larger number of studies on EGFR, which plays an important role in oncogenesis, compared to any average protein. In total, there are 428 candidate proteins that may be PON2 interactors, but almost all of them appeared to have negligible connectivity with high-confidence interactors, with the exception of EGFR. Therefore, it should be noted that the PON2 interactome may be significantly expanded in the future; at present, however, we limited ourselves to creating, for illustrative purposes, a network that includes: a) high-confidence interactions with PARK7 and LRIG1,2; b) interactors that we identified with the help of two-hybrid screening; c) protein hubs that allow us to assess the interconnection of groups “a” and “b” with each other in the global interactome (Fig. 1).

It is important to note that in both cases EGFR becomes included in the PON2 interactome and this may be an indication of the special role of PON2 specifically in EGFR mutated cancer cells. It cannot be ruled out that PON2 interacts with EGFR directly. We should also briefly review the state of knowledge on these new candidates for PON2 interactors. ITM2A, a type 2 integral membrane protein, is a tumor suppressor and its loss increases the aggressiveness of ovarian cancer [39]. ACAA2, a mitochondrial 3-ketoacyl-CoA-thiolase, may play a special role in IDH-mutant gliomas [40]. PPP4R2 (protein phosphatase 4 regulatory subunit 2) is a part of DNA repair complex and plays a role in regulation of sensitivity to platinum drugs [41]. Decorin (DCN) is an important antitumor component of extracellular matrix [42], produced by fibroblasts, whose interaction with EGFR is well studied, moreover, decorin acts as a pan-receptor inhibitor of tyrosine kinases, binding also to HER2, HGFR/Met, VEGFR2, TLR and IGFR [43]. SFRP4 is a secreted protein associated with some aggressive cancers, in case of glioblastoma its action can be proapoptotic [44], it is probably an oncosuppressor, and its mutations reverse this function, which is especially evident in ovarian cancer [45, 46].

Experimentally confirmed is the interaction of PON2 with LRIG1 and LRIG2 proteins, that are ligands of receptor tyrosine kinases and are involved in tumor development. It turned out that through interaction with LRIG1, PON2 affects the expression of another protein, PDGFRA, that is involved in cell proliferation [38]. The interaction of PON2 with PARK7/DJ-1 possibly plays a role in the pathogenesis of some types of Parkinson’s disease, for example, in 1-methyl-4-phenylpyridinium poisoning [37], but PARK7/DJ-1 protein also plays an important role in the resistance of many cancer cell types to antitumor treatments [47].

It can be assumed that both subcellular localization and the interactome of PON2 may differ in cells of various tissues depending on their physiological state. For example, it was found that PON2 interacts with several human immunodeficiency virus-1 and SARS-CoV-2 proteins [48, 49]. Obviously, the interactome can undergo significant perturbations, especially in diverse pathological processes.

Our results, along with other data, point to a significant role of PON2 and its interactors in the aggressiveness of many cancers and their resistance to therapeutic interventions. The diversity of PON2 interactors may also be regarded as evidence for the putative chaperoning function of PON2 as a turnover regulator for many other functionally important proteins [50]. To confirm the role of the putative interactions of PON2 with the identified proteins (decorin, EGFR, etc.) it is necessary to analyze these interactions with both wild-type proteins and mutant variants known in cancer cells.
